# Impact of the Aboral Pouch in Roux-en-Y Reconstruction after Laparoscopic Total Gastrectomy for Elderly Patients

**DOI:** 10.14789/jmj.JMJ23-0036-OA

**Published:** 2024-05-24

**Authors:** AKIRA KUBOTA, SUGURU YAMAUCHI, YUTARO YOSHIMOTO, KENKI TSUDA, YUKINORI YUBE, SANAE KAJI, HAJIME ORITA, MALCOLM V BROCK, TETSU FUKUNAGA

**Affiliations:** 1 Department of Esophageal & Gastroenterological Surgery, Juntendo University Hospital, Tokyo, Japan; 1 Department of Esophageal & Gastroenterological Surgery, Juntendo University Hospital, Tokyo, Japan; 2 Department of Surgery, The Johns Hopkins University School of Medicine, Baltimore, MD, US; 2 Department of Surgery, The Johns Hopkins University School of Medicine, Baltimore, MD, US

**Keywords:** aboral pouches, aged, postgastrectomy syndromes, gastrectomy

## Abstract

**Objectives:**

The number of elderly people with stomach cancer is increasing; therefore, minimally invasive surgical treatments are required. Elderly patients have multiple comorbidities and are prone to postoperative weight loss, nutritional disorders, Postgastrectomy syndrome (PGS), and decreased quality of life (QOL). Total gastrectomy is particularly associated with these complications, although aboral-pouch creation reportedly improves the condition by compensating for lost reservoir capacity. However, there is no consensus regarding its significance. This study aimed to investigate the impact of the aboral pouch on total gastrectomy outcomes in elderly patients.

**Materials and Methods:**

Thirty-six patients who met the eligibility criteria, defined as elderly patients aged ≥75 years, were retrospectively analyzed. The patients had undergone Roux-en-Y reconstructions with an aboral pouch in laparoscopic total gastrectomy procedures performed at Juntendo University from July 2016 to June 2022. The main outcomes were postoperative nutritional status, PGS, and QOL.

**Results:**

The average postoperative period was approximately 1 year (12.0 *vs*. 13.5 months, *P*=0.536), for 14 elderly and 22 non-elderly patients, respectively. Elderly patients had more comorbidities (78.5% *vs*. 40.9%, *P*=0.041). The outcome of nutritional status demonstrated no differences in weight-loss rate (-5.3% *vs*. -8.6%, *P*=0.651) or prognostic nutritional status (-7.9% *vs*. -5.9%, *P*=0.243). There was no significant difference in PGS and QOL between elderly and non-elderly patients.

**Conclusions:**

Total gastrectomy with an aboral-pouch creation could be beneficial for elderly 43 patients from the perspective of postoperative nutritional status, PGS, and QOL.

## Introduction

Total gastrectomy is the gold standard of treatment for upper-stomach cancers. Recently, with the aging of society, the number of elderly individuals requiring surgical treatment for gastric cancer has increased. Many elderly patients who require treatment have multiple comorbidities and are at a higher risk of postoperative complications than their non-elderly counterparts^1-3)^. Furthermore, elderly patients who have undergone gastrectomy procedures have a high weight-loss rate and are more prone to developing severe postoperative nutritional problems^4)^. Postgastrectomy syndrome (PGS), which is specific to gastric surgery, occurs frequently after gastrectomy and requires attention as a clinical condition, because it is closely related to weight loss and nutritional disorders and adversely affects quality of life (QOL)^5-7)^. Specifically, compared with other gastrectomy procedures, total gastrectomy, wherein removal of the entire stomach significantly reduces reservoir capacity, is associated with more frequent weight loss, nutritional disorders, and decreased QOL due to PGS^6, 7)^.

Weight loss after total gastrectomy occurs in approximately 10-15% of cases. Decreased food intake and esophageal reflux symptoms due to bile reflux are particularly well-known causes^5-8)^. Several reconstructive innovations reportedly improve PGS and QOL postoperatively in patients who have undergone total gastrectomy procedures. However, the best practice has not yet been established^8, 9)^.

One method known to improve the reservoir capacity function after total gastrectomy is the addition of a jejunal pouch to the reconstruction. This pouch, created in the Y limb of Roux-en-Y reconstruction, is called the aboral pouch^8, 10, 11)^. Despite several reports on the usefulness of aboral pouches, there is no consensus regarding the safety and benefits of aboral pouches in elderly patients. Herein, we aimed to examine the effect of aboral-pouch creation after laparoscopic total gastrectomy in elderly patients in terms of outcomes such as nutritional status, PGS, and QOL.

## Materials and Methods

### Patients and data collection

**Figure 1:**
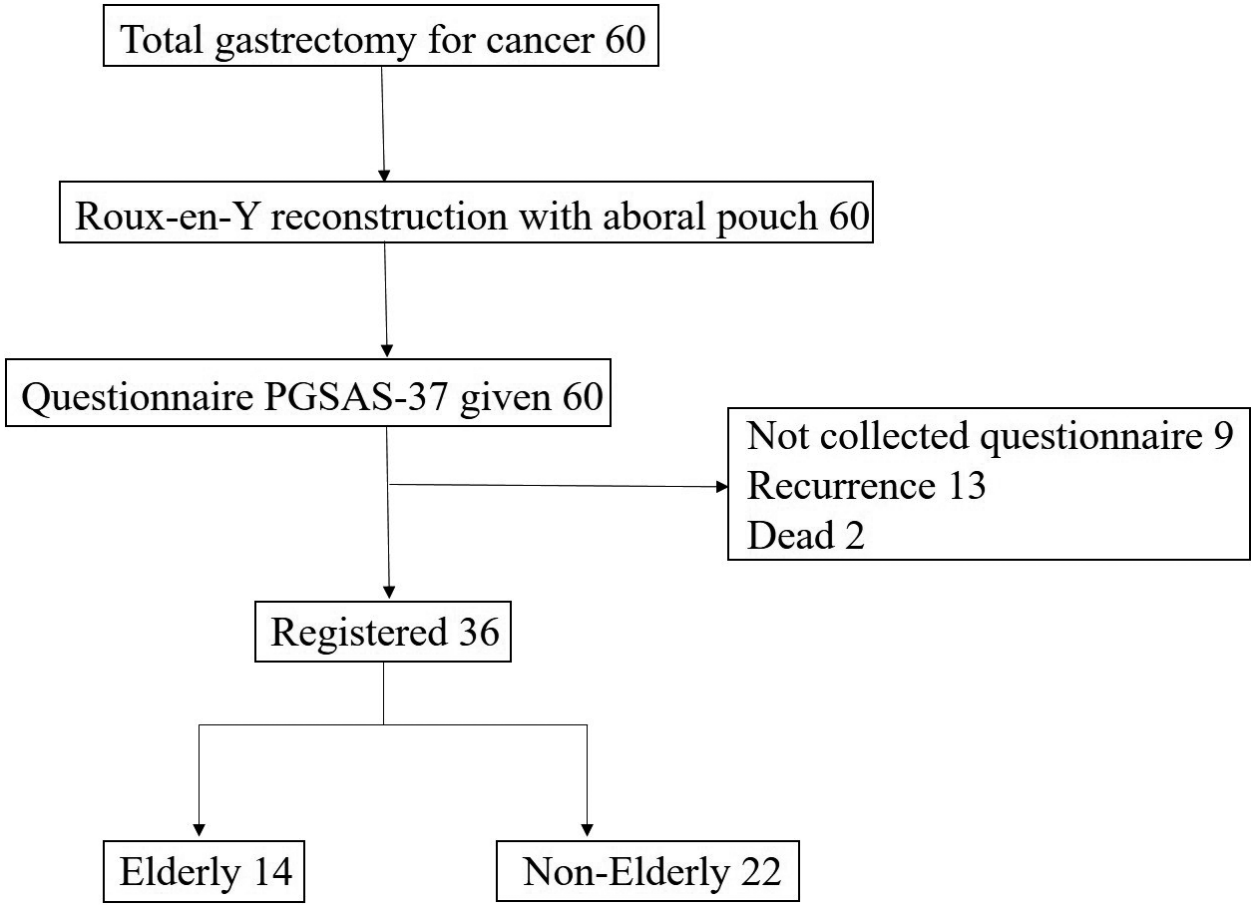
Flow diagram of this study

From July 2016 to June 2020, 60 consecutive gastric cancer patients underwent laparoscopic total gastrectomy at Juntendo University Hospital. Of these, 36 patients in whom R0 resection was obtained, no recurrence occurred, and the Postgastrectomy Syndrome Assessment Scale-37 (PGSAS-37) questionnaire was completed and recorded were included. Herein, elderly patients were defined as those aged ≥75 years. For comparison during analysis, the patients were grouped into those aged ≥75 years (elderly) and <75 years (non-elderly) (Figure 1). Clinical, pathological, perioperative, nutritional status, and the PGSAS-37 questionnaire data were retrospectively evaluated. Clinicopathological data included the postoperative period, sex, body mass index (BMI), comorbidities, lymph-node dissection extent, pathological stage, and combined resection. Perioperative outcomes included surgical approach, conversion to open surgery, operative time, blood-loss volume, postoperative complications, hospital-stay length, and adjuvant chemotherapy. The pathological diagnosis was determined with reference to the Japanese gastric cancer classification^12)^. Postoperative complications were classified using the Clavien-Dindo classification system^13)^. Nutritional status was evaluated based on body weight, total protein, albumin, lymphocyte count, and prognostic nutritional index (PNI)^14)^. The rate of change between 1 year postoperatively and preoperatively was determined.

This study was conducted in accordance with the principles of the Declaration of Helsinki. The study protocol was approved by the Juntendo University Hospital Ethics Review Board according to our clinical ethics regulations (approval no. E22-0412). The requirement for informed consent was waived owing to the retrospective and observational nature of the study. An opt-out approach was used by providing access to written disclosure on the study website (URL: https://www.gcprec.juntendo.ac.jp/kenkyu/detail/5380).

### PGSAS-37

**Figure 2:**
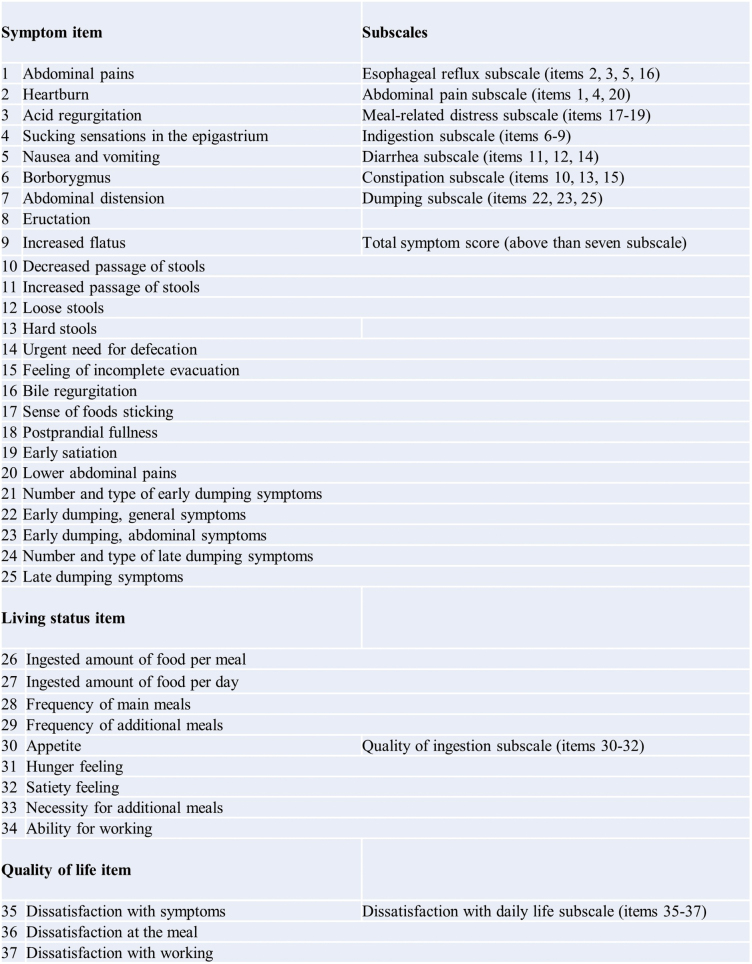
Structure of Postgastrectomy Syndrome Assessment Scale-37

We used the PGSAS-37, established as the national average database in Japan by the Japanese Postgastrectomy Syndrome Working Party (JPGSWP), to compare PGS and QOL in elderly patients with those in non-elderly patients^15, 16)^. The PGSAS-37 is a disease-specific scale used to assess the subjective symptoms and living conditions in patients who have undergone gastrectomy procedures^15)^. PGSAS-37 comprises various QOL questionnaires on the Gastrointestinal Symptom Rating Scale (GSRS)^17)^ and includes 37 items, 15 derived from the GSRS and 22 originally selected as clinically relevant by the JPGSWP. These additional 22 items include 8, 2, 5, 3, and 3 items to assess global symptoms, dumping syndrome, food quantity, food quality, and work status, respectively. Furthermore, it includes one item and three items that rate dissatisfaction with life. These 37 items were aggregated into 9 subscales for 17 primary endpoints. The nine subscales were calculated from the mean values of applicable items. The main outcomes include symptoms, living status, and QOL (Figure 2). High scores for the following items indicated good QOL: food intake, appetite, hunger, satiety, food quality, and bodyweight changes. Low scores for other items indicated a good QOL. The questionnaire was administered to patients at the time of the outpatient visit by a doctor or nurse and was completed in the waiting room by the patient under stress-free conditions. A medical clerk managed data acquisition.

### Aboral pouch

Two types of jejunal pouches are used for Roux-en-Y reconstruction after total gastrectomy. One method involves the creation of a jejunal pouch in the Roux limb of the esophagojejunostomy and the other involves the creation of a jejunal pouch in the Y limb, known as the aboral pouch. Both methods often use a linear stapler to add to the reconstruction. There is no fixed definition of pouch size or method for creating the pouch; however, in practice, a more convenient aboral pouch is likely to be preferred^8, 11, 18)^.

**Figure 3:**
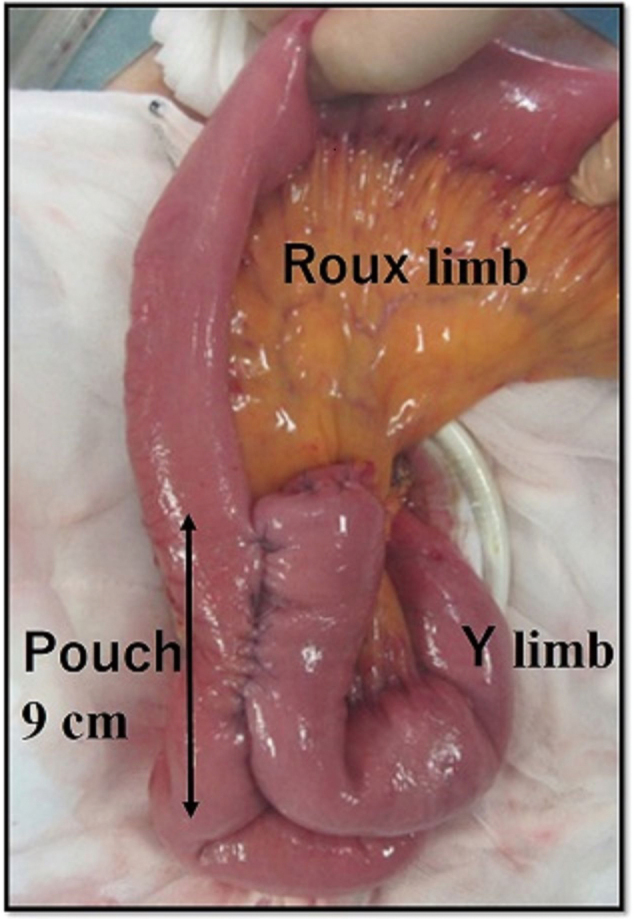
Aboral pouch

The following method was routinely practiced to create aboral pouches: after lymph node dissection and gastrectomy, a small incision was made in the umbilicus. The jejunum was lifted, and an aboral pouch was added to the Y limb, which was created as a side-to-side anastomosis 45 cm distal to the esophagojejunostomy. This procedure for creating a Y-limb with an aboral pouch involved shaping by firing a 60-mm linear stapler from the entry hole of the jejunum to each of the proximal and distal sides. The stapler insertion hole was closed with a handsaw Gambee suture using a braided absorbable suture. Created using two 60-mm linear staplers, the pouch shrinks to approximately 90 mm in length (Figure 3).

### Statistical analysis

We used independent t-tests to compare continuous variables and Fisher's exact test to compare categorical variables. Statistical significance was defined as a two-tailed P-value of <0.05. All statistical analyses were performed using StatMate statistical software (version V; GraphPad Software, San Diego, CA, USA). The PGSAS statistical kit was used to compare PGS and QOL between the elderly and non-elderly groups.

## Results

### Clinicopathological characteristics

Table 1 demonstrates the clinicopathological characteristics of the elderly (≥ 75 years) and non-elderly (< 75 years) groups. There were 14 patients in the elderly group and 22 in the non-elderly group. Patients in the elderly group had significantly more comorbidities than those in the non-elderly group (78.5% *vs.* 40.9%; *P*=0.041). There was no significant difference in the postoperative period recorded in months, sex, preoperative BMI, extent of lymph node dissection, pStage, and combined resection between the two groups.

**Table 1:** Patient clinicopathological characteristics

Variable	Elderly group (n=14)	Non-elderly group (n=22)	Univariable P-value
Age, years, mean (±SD)	79.3 (±4.25)	62.8 (±10.7)	<0.001
Postoperative period in months (±SD)	12.0 (±8.94)	13.5 (±7.96)	0.536
Male, n (%)	8 (57.1)	14 (63.6)	0.738
Preoperative BMI in kg/m^2^ (±SD)	21.1 (±3.3)	22.4 (±4.5)	0.388
Comorbidity, n (%)	11 (78.5)	9 (40.9)	0.041
Extent of lymph node dissection, n			0.441
D1+	2	6	
D2	12	16	
pStage, n			0.255
Ⅰ	4	11	
Ⅱ	3	6	
Ⅲ	7	5	
Combined resection, n (%)	4 (28.5)	3 (13.6)	0.349

BMI, body mass index; SD, standard deviation

### Perioperative outcome

The perioperative outcomes are summarized in Table 2. All patients underwent laparoscopic surgery; for one elderly patient, the procedure was converted to open surgery. There were no significant differences in operative time or intraoperative blood loss. Postoperative complications of Clavien-Dindo grade ≥3 were observed in two (14.3%) and three (13.6 %) patients in the elderly and non-elderly groups, respectively. There were no anastomotic-related complications including the aboral pouch in both groups. The postoperative hospital stay was 17.0 and 13.2 days in the elderly and non-elderly groups, respectively (*P*=0.333).

**Table 2:** Perioperative outcomes

Variable	Elderly group (n=14)	Non-elderly group (n=22)	Univariable *P*-value
Approach, n			1.000
Robotic	1	1	
Laparoscopic	13	21	
Conversion to open surgery, n (%)	1 (7.1)	0	0.389
Operative time, min (±SD)	345 (±114)	359 (±98)	0.172
Blood loss, mL (±SD)	104 (±148)	56.0 (±55.9)	0.705
Postoperative complication CD ≥3, n (%)	2 (14.3)	3 (13.6)	1.000
Pneumoniae, n	1	0	
Surgical site infection, n	1	3	
Anastomotic-related complication including the aboral pouch	0	0	
Postoperative hospital stay in days (±SD)	17 (±71.1)	13 (±19.7)	0.333

CD, Clavien-Dindo; SD, standard deviation

### Nutritional Status

The nutritional status is summarized in Table 3. There was no significant difference in weight loss between the elderly and non-elderly groups (-5.3% *vs*. -8.6%, *P*=0.651). There was no significant difference in total protein, albumin, total lymphocyte count, or PNI (-7.9% in the elderly group *vs*. -5.9% in the non-elderly group, *P*=0.243) between the two groups.

**Table 3:** Nutritional status

Variable	Elderly group (n=14)	Non-elderly group (n=22)	Univariable *P*-value
Body weight % (±SD)	-5.3 % (±20.0)	-8.6 % (±23.8)	0.666
Total protein % (±SD)	3.7 % (±30.1)	-0.2 % (±10.3)	0.585
Albumin % (±SD)	-7.4 % (±43.0)	3.2 % (±44.5)	0.389
Total lymphocyte count % (±SD)	-7.5 % (±42.1)	1.3 % (±31.7)	0.476
PNI % (±SD)	-7.9 % (±38.2)	-5.9 % (±31.4)	0.243

PNI, prognostic nutritional index; SD, standard deviation Nutritional Status (%): (1 year postoperatively-preoperatively)/preoperatively × 100

### PGS and QOL

No significant differences in symptom categories were observed between the elderly and non-elderly groups (Table 4). Furthermore, no significant differences in living status or QOL were observed between the two groups (Table 5).

**Table 4:** PGSAS score for symptom categories

		Elderly group n=14		Non-elderly group n=24		*P*-value
		mean	SD	mean	SD	
Symptom	Esophageal reflux subscale	1.9	0.8	2.5	1.0	0.074
	Abdominal pain subscale	2.5	1.2	3.2	1.2	0.091
	Meal-related distress subscale	1.5	0.9	1.7	0.8	0.469
	Indigestion subscale	2.1	0.9	2.3	0.6	0.464
	Diarrhea subscale	2.2	1.9	1.9	1.3	0.604
	Constipation subscale	2.4	1.2	1.9	1.9	0.327
	Dumping subscale	1.7	1.0	1.9	1.9	0.674
	Total symptoms score	2.1	0.7	2.1	0.5	1.000

PGSAS, Postgastrectomy Syndrome Assessment Scale

**Table 5:** PGSAS score for living status and QOL categories

		Elderly group n=14		Non-elderly group n=24		*P*-value
		mean	SD	mean	SD	
Living status	Change in body weight (%)	-5.3	20.0	-8.6	23.8	0.651
	Amount of food ingested per meal (%)	6.2	2.2	6.0	1.1	0.752
	Necessity of additional meals	2.1	0.5	2.1	0.8	1.000
	Quality of ingestion subscale	2.6	1.1	2.6	1.4	1.000
	Ability for working	2.9	1.3	2.1	1.1	0.061
QOL	Dissatisfaction with symptoms	1.7	0.9	1.7	0.8	1.000
	Dissatisfaction during meals	2.4	1.5	2.1	1.1	0.518
	Dissatisfaction during work	2.0	1.2	1.8	0.8	0.582
	Dissatisfaction with daily life subscale	1.9	1.1	1.9	1.9	1.000

PGSAS, Postgastrectomy Syndrome Assessment Scale; QOL, quality of life

## Discussion

This is the first report to evaluate the aboral-pouch addition to Roux-en-Y reconstruction after laparoscopic total gastrectomy procedure in elderly patients. This study is significant because it evaluated aboral-pouch addition from multiple perspectives, including perioperative outcomes, postoperative nutritional status, PGS, and QOL, and demonstrated its safety and feasibility in elderly patients with gastric cancer for whom total gastrectomy is indicated.

The core treatment for gastric cancer is surgical resection. Total gastrectomy is required for curative resection of cancers occupying the upper part of the stomach. Roux-en-Y is the most common type of reconstruction performed after total gastrectomy globally^19)^. However, reservoir capacity reduction due to total gastrectomy is the main cause of poor nutritional status, PGS, and QOL post-procedure^1)^. Recently, the number of elderly patients requiring total gastrectomy has been increasing due to the aging population. These elderly patients are at high risk for postoperative complications, nutritional disorders, PGS, and reduced QOL post- operatively^2-4)^. Therefore, we focused on aboral-pouch addition to total gastrectomy procedures with Roux-en-Y reconstruction in elderly patients.

According to several studies, there is 10-15% weight loss after total gastrectomy procedures, especially in elderly patients, which is a serious concern that needs to be addressed^1, 5, 8)^. The current study indicated no significant difference in the rate of postoperative weight loss between the elderly and non-elderly groups, although the rate of weight loss tended to be lower in the elderly group. We hypothesized that this was because the elderly group tended to have a lower preoperative BMI, so there was no significant difference in weight loss rate between the two groups, although it is said that elderly patients tended to be higher weight loss after surgery because they have various comorbidities and lack reserve capacity. Furthermore, the 1-year rate of change in serum total protein, albumin, and total lymphocyte count (commonly used as nutritional indices after gastrectomy) and PNI (demonstrated to be useful for gastrointestinal surgery) was comparable with that in the non-elderly group. These findings suggest that aboral-pouch addition in Roux-en-Y reconstruction after laparoscopic total gastrectomy is beneficial for elderly patients diagnosed with gastric cancer. However, this result may not be solely due to aboral-pouch addition during surgery but also because patients have been receiving postoperative nutritional guidance, medications, life guidance, and psychiatric care. This study demonstrated that aboral-pouch addition did not cause short-term complications or adverse nutritional effects. These results are similar to those reported previously, where an aboral pouch was proposed to compensate for reduced reservoir capacity. Nutritional indicators, such as total protein, were maintained, which would support such results in higher-risk elderly patients^20, 21)^.

Tsuji et al. reported that the surgical technique required for aboral-pouch addition to Roux-en-Y reconstruction was not complicated and could be safely performed as the stapler used for reconstruction has evolved from two to three rows^8)^. However, we postulate that aboral-pouch creation after total gastrectomy has not become a standard technique partly owing to surgeons’ concerns regarding associated complications. Indeed, complications, although infrequent, have been reported, including obstruction due to excessive pouch expansion and pouch necrosis^22, 23, 24)^. Our results showed no complications related to the anastomotic site including the aboral pouch. The observation period of this study was approximately 1 year; further follow-up is required to determine possible long-term complications.

We created the Y limb after total gastrectomy extracorporeally because of its maneuverability and simplicity. Although there is an additional cost for a linear stapler, we only used a 60-mm stapler to create the pouch when making the Y-limb. No special, complicated technique was required, and the operative time was not prolonged. The patients in our study did not experience any intraoperative or postoperative aboral pouch-related complications. Hence, this technique should not be avoided on the basis of complications and surgical technique-related issues.

The difficulty in assessing clinical effectiveness, including parameters such as PGS and QOL, after gastrectomy is due to variability in assessment metrics. The Short Form Health Survey and GSRS are useful but cannot evaluate dumping symptoms or specific meal-related symptoms that occur frequently after gastrectomy^25, 26)^. EORTC QLQ- C30 and STO-22 have also been developed for the evaluation of QOL in cancer patients undergoing treatment. However, these are not suitable for the assessment of several essential symptoms of PGS^27, 28)^. PGSAS-37 can comprehensively evaluate PGS and QOL using a self-reported questionnaire for gastric cancer patients who have undergone gastrectomy procedures^7)^. This questionnaire contains questions about well-known, specific, and characteristic symptoms that significantly affect the QOL in patients undergoing gastrectomy procedures. Therefore, herein, the PGSAS-37 was used to evaluate PGS and QOL in patients with an aboral pouch created in laparoscopic total gastrectomy Roux-en-Y reconstruction. There were no worse outcomes reported in the elderly patients compared with the non-elderly patients, including esophageal reflux symptoms and various QOL issues that are more likely to occur in elderly patients. Several studies have reported the evaluation of PGS and QOL with aboral-pouch addition after total gastrectomy. Syn *et al.* reported few dumping and esophageal reflux symptoms, large amounts of food intake, and nutritional superiority^29)^. Alternatively, Tanaka *et al.* conducted a prospective multicenter observational study of 5-year QOL and nutritional status in patients who underwent abdominal pouch reconstruction after total gastrectomy for gastric cancer and reported no significant aboral pouch-associated complaints other than diarrhea, nutritional indices, and QOL^11)^. Although meta-analyses have been conducted on the aboral pouch, there is a possibility that PGS and QOL were not properly assessed. Tsuji *et al* conducted a nationwide multi-institutional cross- sectional study, which suggested that total gastrectomy with the addition of aboral pouch, particularly oral pouches, significantly improved postoperative QOL^8, 30)^. These reports have led to the coverage of aboral-pouch creation in total gastrectomy by insurance in Japan since April 2022. Consequently, the number of surgeries wherein an aboral pouch is added after gastrectomy is expected to increase in the future. Hence, appropriate postoperative functional assessment of these surgeries is an important issue.

This study has some limitations. First, this was a single-center retrospective observational study with a small sample size. Recently, however, procedures to leave a small remnant stomach and proximal gastrectomy have become popular, and cases of total gastrectomy are decreasing. Under such circumstances, a small number of cases from a single-center would be acceptable. We believe that our data are significant because total gastrectomy is rare. Second, this study assessed outcomes 1 year after total gastrectomy procedures, and long-term follow-up is necessary. However, the nutritional indices, PGS, and QOL investigated herein stabilized at 1 year postoperatively; therefore, no major problems should be expected during the observation period^5)^. Third, it was difficult to rationally explain all findings observed. PGS and QOL vary widely among individuals and are influenced by various physical and functional factors. The aboral pouch for Roux-en-Y reconstruction in total gastrectomy is created either on the Roux limb or on the Y limb. However, creation on the Y limb is more common, and our institution does not create an aboral pouch on the Roux limb (oral side). Hence, this was not the subject of the study. Further research is required to determine the appropriate site for pouch creation and the appropriate pouch size.

Total gastrectomy with an aboral Roux-en-Y pouch could be beneficial for elderly patients in terms of postoperative nutritional status, PGS, and QOL.

## Funding

No funding was received.

## Author contributions

AK contributed to the study concept and design; data acquisition, analysis, and interpretation; drafting the manuscript; and performed the statistical analysis. SY contributed to the study concept and design; data analysis and interpretation; and manuscript revision. YutY, KT, YukY, and SK performed data acquisition. HO, MVB, and TF supervised the study. All authors read and approved the final manuscript.

## Conflicts of interest statement

The authors declare that there are no conflicts of interest.

## Availability of data and materials

The authors confirm that data supporting the findings of this study are available within the article.
